# Dr. Ferriar, on Hull's Essay on Phlegmatia Dolens

**Published:** 1801-01

**Authors:** John Ferriar

**Affiliations:** Manchester


### *To* Dr. BATTY.

DEAR SIR,

I Conceive that your valuable Journal ſerves, among other uſeful purpoſes, as a Court of Honour, to which any Member of the Faculty may appeal, reſpecting the miſconduct of another. Allow me, therefore, through this channel, to point out to the cenſure of all profeſſional gentlemen, a very unuſual ſpecies of attack, which has been made upon me in a late publication.

The book to which I allude, is an Eſſay on *Phlegmatia Dolens*, written by Dr. Hull, of this place; in the courſe of which he has undertaken to controvert my Eſſay *On a Diſeaſe of the Lymphatic Veſſels, hitherto miſunderſtood*, which was inſerted in the third volume of my Medical Hiſtories and Reflections. The works of every author are open to criticiſm, and I have never betrayed impatience when objections have been fairly and candidly candidly made againſt any part of mine. The public opinion concerning my writings is already formed; and it would have given me no concern, that they were diſapproved by pedants without learning, and compilers without diſcernment. But the offence which I mean to denounce is of a moſt ſerious nature.

In my Eſſay, which was publiſhed in 1798, I had introduced an account of the caſe of Jane Waters, a home-patient of the Mancheſter Infirmary, who had been recently under my care, and I mentioned that it was an accurate ſtatement. I have lately ſeen with aſtoniſhment, in the Eſſay on *Phlegmatia Dolens*, that Dr. Hull, who has no connection with the Infirmary, or intercourſe with me, *has called upon this woman*, for the purpoſe of correcting my account of her caſe! And that, from the confuſed recollection of this poor ignorant creature, at the diſtance of *nearly two years* from the occurrence of the diſeaſe, he has had the preſumption to publiſh a ſtatement of her complaint, contradicting mine in ſeveral important particulars! With a man who ſhows a total diſregard of profeſſional character, by the avowal of ſuch conduct, I cannot ſtoop to a controverſy.—Give me leave to addreſs ſome ſhort obſervations to your readers.

My reports of this caſe, as of many others, were drawn from notes which I took at every viſit I paid to the patient.—They ſhew the regular progreſs to a cure of the lymphatic inflammation, from the 3d to the 9th of January, 1798; and of an inflammatory affection of the knee, which was excited by her imprudent exertions, about the 16th of that month. My report, on the 21ſt of January, runs thus: "The affection of the limb is completely removed. She has no complaint, excepting debility, and a cough, which ſhe had contracted previouſly to her lying-in." On referring to the liſt of home-patients,* I find that Jane Waters was made an out-patient on the 27th of the ſame month.† In oppoſition to theſe facts, Dr. Hull has produced a narrative without ſome eſſential dates, and, I am ſorry to ſay, without probability.—Whatever ſubſequent diſeaſe the patient might experience, the evidence of the home-patient-

* In this liſt, which is kept in ſeparate books by the phyſician's clerk, are ſpecified the name and age of the patients, the place of their abode, the name of the diſeaſe, the names of the recommender, the number of viſits, which are diſtinguiſhed as they are made by the phyſician or the clerk, and the time and manner of diſcharging the patients.—The value of theſe records has been often felt.

† Home-patients of the Mancheſter Infirmary are thoſe who are attended at their own houſes.—Out-patients are thoſe who are able to come for their medicines to the Diſpenſary on ſtated days, as in ſimilar inſtitutions.

patient-book muſt prove, that ſhe was able to walk abroad on the 27th of January, 1798.

I ſhall take this opportunity of obſerving, that I have received ſome very important facts reſpecting lymphatic inflammation, from ſeveral reſpectable practitioners, ſince the publication of my third volume.—Theſe ſhall be laid before the public as ſoon as my profeſſional avocations will permit.—They will confirm two points of my Eſſay, in which I have made an addition to the hiſtory of the diſeaſe: 1ſt. In obſerving the inflamed and enlarged ſtate of the trunks of the lymphatic veſſels; 2d. In ſhewing that it may take place in either ſex, conſequently independent of the circumſtances of parturition.—Mr. Trye's book, which has been mentioned as the ſource of my opinion, I have never read. I underſtood, however, at the time of publiſhing my obſervations, that he confined his views entirely to the diſeaſe in the puerperal ſtate. But it muſt appear to every candid perſon, that by diſcovering the actual enlargement, and inflammation of the lymphatic trunks, in this diſeaſe, I have eſtabliſhed that as a theory, which was before only a plauſible hypotheſis. I derived my opinion from the evidence of my ſenſes, without obligation to any preceding writer, and my method of cure was directly deduced from that opinion.

It is with great reluctance that I thus come forward in my own vindication.—If it ſhall appear from this narrative, that the author of whom I complain has covered himſelf with diſgrace in the eyes of every man of honour, by his attempt to injure me, the fault is not mine. I am,

Dear Sir,

Your faithful and obedient ſervant,

JOHN FERRIAR.

